# Seasonal Variations in Surface Metabolite Composition of *Fucus vesiculosus* and *Fucus serratus* from the Baltic Sea

**DOI:** 10.1371/journal.pone.0168196

**Published:** 2016-12-13

**Authors:** Esther Rickert, Martin Wahl, Heike Link, Hannes Richter, Georg Pohnert

**Affiliations:** 1 Department of Marine Ecology, Division of Benthic Ecology, Helmholtz Centre for Ocean Research Kiel GEOMAR, Kiel, Germany; 2 Institute for Ecosystem Research, Kiel University, Kiel, Germany; 3 Institute for Inorganic and Analytical Chemistry, Department for Bioorganic Analytics, Friedrich Schiller University Jena, Jena, Germany; University of Sydney, AUSTRALIA

## Abstract

Perennial macroalgae within the genus *Fucus* are known to exude metabolites through their outer thallus surface. Some of these metabolites have pro- and/or antifouling properties. Seasonal fluctuations of natural fouling pressure and chemical fouling control strength against micro- and macrofoulers have previously been observed in *Fucus*, suggesting that control strength varies with threat. To date, a study on the seasonal composition of surface associated metabolites, responsible for much of the fouling control, has not been done. We sampled individuals of the two co-occurring species *F*. *vesiculosus* and *F*. *serratus* at monthly intervals (six per species and month) during a one-year field study. We analysed the chemical composition of surface associated metabolites of both *Fucus* species by means of gas chromatography-mass spectrometry (GC-MS) to describe temporal patterns in chemical surface composition. Additionally, we correlated abiotic and biotic parameters recorded monthly within the sampled habitat with the variation in the chemical surface landscape of *Fucus*. Our study revealed that the chemical surface composition of both *Fucus* species exhibits substantial seasonal differences between spring/summer and autumn/winter months. Light and temperature explained most of the seasonal variability in surface metabolite composition of both *Fucus* species. A strong summerly up-regulation of eighteen saccharides and two hydroxy acids in *F*. *vesiculosus* as well as of four fatty acids and two saccharides in *F*. *serratus* was observed. We discuss how these up-regulated molecules may have a complex effect on associated microfoulers, both promoting or decreasing fouling depending on metabolite and bacterial identity. These seasonal shifts in the surface metabolome seem to exert a compound control of density and composition of the *Fucus* associated biofilm.

## Introduction

Macroalgal surfaces function as an interface with the aquatic environment. All essential physiological processes such as light absorption, gas exchange, nutrient uptake or the release of metabolic products take place via this interface [1]. As physiologically highly active interfaces, macroalgal thallus surfaces are often enriched with released photosynthesis products such as oxygen and carbohydrates [2–4]. Besides these photosynthesis products a variety of different metabolites have been detected in the immediate vicinity of macroalgal surfaces. For example, polyhalogenated and polyphenolic compounds were found at or near the surface of red and brown macroalgae [5–7]. Furthermore, the pigment fucoxanthin, the osmolyte dimethylsulphopropionate (DMSP) as well as the amino acid proline have been detected on the surface of the brown macroalgae *Fucus vesiculosus* [8–10]. Such surface-associated metabolites are also referred to as “surface metabolites”. We know that halogenated furanones can be released by the red macroalgae *Delisea pulchra* from so-called gland cells located under the thallus surface [11]. However, the origin or transport mechanisms for the majority of known surface metabolites are not yet understood.

Macroalgal surfaces are exposed to a diverse and seasonally variable prokaryotic fouling pressure and are typically colonized by up to 10^7^–10^8^ bacteria cells per cm^2^ of thallus, depending on the algal species [12–14]. Uncontrolled microbial fouling would entail a reduction of incoming light [15] as well as a reduced gas and nutrient exchange, resulting in a lower photosynthesis efficiency (as described for epiphytes on seagrass; [16, 17]). Several studies have demonstrated that macroalgae use exuded metabolites in order to prevent or regulate bacterial attachment, growth and, hence the density of associated bacteria [9, 10, 18, 19]. Furthermore, it has been shown that different macroalgal metabolites can shape the composition of the bacterial community composition [20–23].

Since macroalgae are photosynthetic organisms, their metabolism strongly depends on environmental parameters such as light and temperature, as well as on the availability of nutrients [24–27]. It has been shown that the tissue content as well as the exudation rates of polyphenols and carbohydrates vary in response to environmental parameters for some species of brown algae [2, 7, 28, 29]. Additionally, it is known that the strength of chemical defence or even specific antifouling metabolites against bacteria of different species of macroalgae exhibit seasonal variations, showing a general up-regulation during summer months when metabolic rates and fouling pressure are high [30–33]. As fouling pressure as well as resource availability vary during the year, especially in temperate regions, it could be expected that macroalgae also exhibit a synchronised anti-bacterial defence strength in such a fluctuating environment. A simultaneous assessment of the temporal patterns of environmental variables, fouling pressure, and the chemical landscape at the thallus surface through all seasons has not been undertaken before.

The present study focuses on the perennial brown macroalgae *F*. *vesiculosus* and *F*. *serratus* from the temperate Baltic Sea. Former investigations mainly focused on the chemical fouling control of *F*. *vesiculosus* [9, 10, 17, 20], whereas the chemical fouling control of the closely related *F*. *serratus* has received little attention, so far. To deepen the knowledge about the chemical fouling control of *Fucus* it is of importance to investigate further species of this genus. *Fucus vesiculosus* and *Fucus serratus* have been used as study organisms in the present investigation since they are the dominant *Fucus* species in the study area, representing the ecological important genus *Fucus*.

To date, only little is known about the seasonal variation of *Fucus* surface metabolites and how environmental parameters such as light, temperature, nutrients and prokaryotic fouling pressure influence this chemical boundary layer. In-depth knowledge regarding the chemical composition of *Fucus* surface metabolites and their seasonal patterns is essential to gain a better understanding of the chemical fouling control in this genus.

The aim of the present study was to investigate the seasonal variation in surface metabolite composition of *F*. *vesiculosus* and *F*. *serratus* and how the metabolite composition relates to the seasonal variations in the environmental factors light, temperature, nutrients and prokaryotic fouling pressure. This study was conducted simultaneously with a study analysing seasonal fluctuations in chemical control against macro- and microfouling, where data on the environmental parameters and prokaryotic fouling data have been published [34, 35]. The following questions structured our project on seasonal variation in surface metabolite composition: (I) Are there significant differences in the surface chemistry composition of *Fucus* between different seasons? (II) Which metabolites contribute most to the seasonal differences in surface chemistry? (III) Which abiotic and biotic parameters correlate significantly with the seasonal shifts in metabolite composition of *Fucus*?

## Material and Methods

### Sampling of algal material

The two perennial brown macroalgae *Fucus vesiculosus* Linnaeus (1753) *and Fucus serratus* Linnaeus (1753) were sampled monthly over an entire year (August 2012—July 2013) at Bülk, outer Kiel Fjord, Germany (54°27’21 N / 10°11’57 E). *F*. *vesiculosus* and *F*. *serratus* occupy overlapping horizons here, with the former ranging from 0 to 2 m and the latter from 0.5 to 3 m depth. Six non-fertile *Fucus* individuals per species and per month (*n* = 18 per season) were collected from mixed stands at a depth of 0.5 m under mid water level. Transportation to the laboratory took place in 3 l plastic bags and a cooler box to avoid desiccation and temperature stress.

Specific field permission was not required to perform the field experiment in Bülk, outer Kiel Fjord, Germany. The field study did not involve endangered or protected species.

### Environmental parameters and fouling pressure

Data loggers (*n* = 3; HOBO UA-002-64, Onset Computer Corporation, Bourne, Massachusetts, USA) were deployed at a depth of 0.5 m under mid water level within the mixed *Fucus* stands at the sampling site and temperature and light were recorded hourly. Water samples from the same depths were taken weekly and analysed for nitrogen (nitrate + nitrite, ammonium) and phosphate concentrations. These environmental parameters were recorded during a study that ran simultaneously to the one presented here and have previously been published [34]. Data are available at the public data repository 'PANGAEA Data Publisher for Earth & Environmental Science' (doi:10.1594/PANGAEA.858055). For detailed method descriptions see [34].

Briefly, to assess the relative seasonal variation in prokaryotic fouling pressure at the sampling site, horizontally oriented microscope slides (*n* = 9) were exposed at a depth of 0.5 m under mid water level for seven days each month. After retrieval, the slides were fixed in 3.7% formaldehyde solution at 4°C overnight and subsequently rinsed with sterile filtered 1x phosphate-buffered saline (PBS) solution, then stored in a PBS-ethanol solution (1:1 v/v of 1x PBS and 96% ethanol) at -20°C until further sample processing. Approx. 1 cm^2^ of the microscopy slides was stained with 10 μl of a ready-to-use DAPI (4’.6-diamidino-2-phenylindole) containing mounting medium (Roti®-Mount FluorCare DAPI, Roth, Karlsruhe, Germany) and covered with a cover glass. For prokaryotic cell enumeration, five randomly selected visual fields per replicate were photographed (epifluorescence microscope: Axio Scope.A1, Carl Zeiss Microscopy GmbH, Göttingen, Germany; camera: ProgRes^®^ CF, Jenoptik, Jena, Germany). Photos were manually analysed by counting all prokaryotic cells in 20 randomly selected squares (each 50 μm^2^). The relative seasonal variation in prokaryotic fouling pressure was recorded during a study that ran simultaneously and has previously been published [35]. Data are available at the public data repository 'PANGAEA Data Publisher for Earth & Environmental Science' (doi:10.1594/PANGAEA.864067).

### Surface extraction

Eighteen *Fucus* individuals per species and season (six per month) were surface-extracted. Per alga individual, approx. 50 g of the upper 5–10 cm apical thalli tips devoid of macrofoulers were cut off. 1 g (wet weight) of *F*. *vesiculosus* thallus material corresponds approx. to a surface area of 25.57 cm^2^ [13]. The surface extraction of *Fucus* was performed according to the protocol of de Nys and Dworjanyn [36] with minor modifications (see below). Before extraction, the thalli tips were spin dried in a salad spinner for 30 s to remove excess seawater from the alga material. The extraction time was set to 4 s in order to minimize the risk of epidermis damage and extraction of internal metabolites. For details on the extraction procedure see [34]. For the extraction, 3–6 thallus tips (depending on size and branching) were dipped into 100 ml of a constantly stirred *n*-hexane and methanol (1:1 v/v) emulsion for 4 s. Careful attention was paid to ensure that the cut surface had no contact with the solvents so as to avoid any leaching of internal metabolites. The surface extractions were performed within 3 to 4 hours after algae sampling. The extracts were filtered with a paper filter (MN 615 ¼, Ø 150 mm, Macherey-Nagel, Düren, Germany) in order to remove particles. The filtered extracts were evaporated at 35°C under vacuum with a rotation evaporator. The reduced extracts were re-dissolved with 2 ml *n*-hexane and methanol, respectively (1:1 v/v). The extracts were dried at 35°C under constant nitrogen flow and stored at -20°C until gas chromatography–mass spectrometry (GC-MS) sample preparation. The entire extraction procedure was carried out under indoor light and temperature conditions.

Solvent blanks (*n* = 4) for GC-MS analysis were prepared by performing the whole extraction procedure without algae material.

### GC-MS sample preparation and analysis

Dry *Fucus* extracts were re-dissolved, first using 2x 800 μl of heptane (≥ 99.9% GC grade, Sigma-Aldrich Chemie Gmbh, Munich, Germany) per extract, followed by 1 min of vortexing and transfer to a new vial for GC-MS. The remaining solid crust of un-dissolved extracts were treated with 2x 800 μl of methanol (≥ 99.9% GC grade, Sigma-Aldrich Chemie Gmbh, Munich, Germany) and 1 min of vortexing to complete the dissolving process. Respectively 40 μl of the extracts solved with heptane and methanol were combined and 2 μl of ribitol internal standard solution (0.4 mM in water, Sigma-Aldrich, Germany) were added, followed by evaporation to dryness under vacuum for ~ 3 h.

Sample derivatisation was performed according to the protocol by Vidoudez and Pohnert [37]. For derivatisation, 50 μl of a freshly prepared methoxymation solution (20 mg methoxyamine hydrochloride, Sigma-Aldrich Chemie Gmbh, Munich, Germany, dissolved in 1 ml of pyridine) were added to the sample followed by 1 min of vortexing. Samples were first incubated at 60°C for 1 h, followed by a second incubation step at room temperature for 9 h. Silylation solution was freshly prepared by adding 40 μl of retention time index mix (Sigma-Aldrich Chemie Gmbh, Munich, Germany) into a fresh vial of *N*-methyl-*N*-(trimethylsilyl) trifluoroacetamide (MSTFA, 1 ml aliquots, Macherey-Nagel, Düren, Germany) with a glass syringe. 50 μl of this silylation solution were added to the sample with a glass syringe and incubated at 40°C for 1 h. Solvent blank samples were prepared for GC-MS analysis in the same way as extract samples (but without the algal extracts). After incubation, samples were transferred into vials with glass inserts and analysed with a GCT Premier TOF mass spectrometer (Waters / Micromass, Manchester, UK). The DB-5ms column had a length of 30 m attached to a 5.7 m pre-column, the source temperature was set to 250°C and the split to 4. The oven temperature was held at 75°C for 3 min, increased with 12°C/min to 315°C and held at that temperature for 7 min. Mass spectra were obtained with 10 scans/sec [37].

### GC-MS data processing

Chromatogram deconvolution was performed using AMDIS 2.71 with a smoothing window of 5 scans and peak integration using MET-IDEA 2.08 with a lower mass limit of 50.

Data of each GC-MS extract measured were corrected to the internal standard ribitol by dividing integrals from extracts by the respective ribitol integrals. In addition, ribitol-corrected data were further corrected by the data of the solvent blanks, in order to avoid analysing readings of contaminants. For blank correction, each data set was subtracted by the mean of solvent blanks (*n* = 4). All negative values were converted to zero after ribitol and solvent blank correction.

### Identification of metabolites

Unless otherwise indicated, peaks were tentatively identified with the spectral library NIST 2011.

### Statistical analysis

A direct comparison between the chemical landscapes found at the thallus surfaces of the two *Fucus* species was not the goal of this study. Consequently, all statistical analyses were run separately for each of the two species. In order to test for significant differences among seasons in the metabolite composition of *Fucus* surface extracts, an analysis of similarity (1-way ANOSIM) was performed. Analyses were based on square root transformed GC-MS data (intensity of respective masses). On the basis of these data, the related resemblance matrix (Bray-Curtis similarity) was calculated for all samples. The factor 'season' (4 levels: spring, summer, autumn, winter) with *n* = 18 replicates per season (exceptions in the *F*. *vesiculosus* data set: spring *n* = 17 and summer *n* = 15) was tested. Classification of the factor ‘season’ was performed according to the meteorological seasons for the northern hemisphere (Dec., Jan., Feb. = winter; Mar., Apr., May = spring; Jun., Jul., Aug. = summer; Sep., Oct., Nov. = autumn). A metric multi-dimensional scaling (MDS) plot was generated to visualize the resulting similarity/dissimilarity patterns. Global-R statistics were used to test for significant differences between groups. R-values ranged from 0 to 1, where high values indicated a large multi-variate dissimilarity among seasons. R-values of > 0.25 showed that the patterns were not random [38].

To assess the relationship between the variation of *Fucus* surface chemistry and the environmental variables (temperature, light, nitrogen, phosphate and prokaryotic fouling pressure), a distance-based linear model (DistLM) was performed [39]. With this procedure, we first tested if there were any significant correlations between the multivariate *Fucus* surface chemistry and each of the environmental variables (marginal tests). In the next steps, the DistLM procedure ran through all variable combinations to identify, which set explains the patterns in the *Fucus* surface chemistry data best (sequential tests).

Prior to running DistLM, data sets were prepared as followed: To match the environmental variable matrix (one replicate per month), the data resemblance matrices containing the square root transformed *Fucus* surface chemistry data (GC-MS data, based on six replicates per month) were converted to a centroid resemblance matrix (Bray-Curtis similarities) based on the factor month. The environmental variable data were normalised and selected as predictor variables. The conversion of the *Fucus* chemistry data into a centroid resemblance matrix was necessary to match the chemistry matrix with the environmental variable matrix. Thus, both matrices had the same sample size (*n* = 12, month). The following DistLM settings were used: stepwise selection, adjusted R^2^ criterion and 9999 permutations.

To analyse which masses, i.e. molecules, were most strongly up- or down regulated in winter and summer surface extracts, a SIMPER routine (similarity percentage analysis) was performed by comparing the winter (*n* = 18) and summer (*n* = 18; *n* = 15 for *F*. *vesiculosus*) GC-MS measured values (masses) based on square root transformed values. All masses cumulatively contributing to 75% of the observed differences were selected from the SIMPER result table. To standardize the response strength, i.e. the relative amount of up- and down-regulation between seasons, first the log of the ratio between the GC-MS masses in summer and winter extracts was calculated from each mass. Secondly, the detected masses ratios were ranked according to their log values with a cut off at 0.7 corresponding to a five-fold increase in summer relative to winter (see ration summer/winter).

All multivariate analyses were performed using the software package Plymouth Routines in Multivariate Ecological Research (PRIMER) version 6 and PERMANOVA+ add-on [38, 39].

## Results

### Seasonal variability of *Fucus* surface chemistry

**Fig 1:**
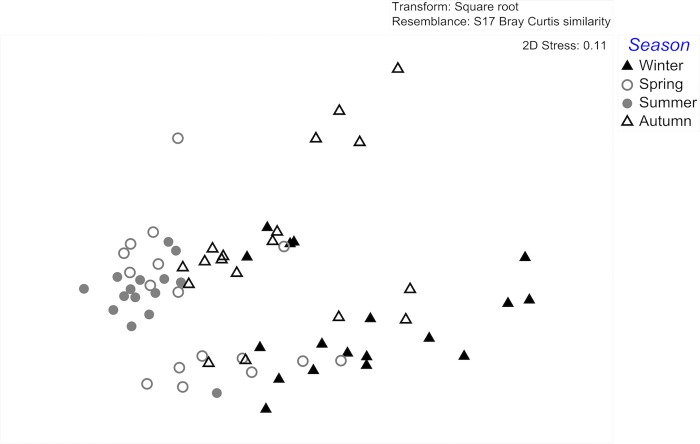
MDS (multi-dimensional scaling) plot of the variance/similarity in *Fucus vesiculosus* surface extract composition originating from different seasons. Symbols represent single monthly samples of *F*. *vesiculosus* individuals within the four seasons (*n* = 18 per season; exceptions: spring *n* = 17, summer *n* = 15).

The chemical composition of *Fucus vesiculosus* surface extracts differed significantly among seasons (ANOSIM global test: global R = 0.342, *p* = 0.0001). The composition of *F*. *vesiculosus* surface extracts sampled in winter differed significantly from surface extracts sampled in spring (ANOSIM pairwise tests: winter/spring R statistic = 0.399, *p* = 0.0001) and summer (ANOSIM pairwise tests: winter/summer R statistic = 0.72, *p* = 0.0001). Summer extracts differed significantly from autumn extracts (ANOSIM pairwise tests: summer/autumn R statistic = 0.346, *p* = 0.0001) (Table 1 and Fig 1).

**Table 1:** Pairwise test results (ANOSIM) for *Fucus vesiculosus* chemical composition of surface extracts.

Groups	R statistic	p-value	Significance level %
**Winter, Spring**	**0.399**	**0.0001**	**0.01**
**Winter, Summer**	**0.72**	**0.0001**	**0.01**
Winter, Autumn	0.239	0.0006	0.06
Spring, Summer	0.161	0.004	0.4
Spring, Autumn	0.231	0.0002	0.02
**Summer, Autumn**	**0.346**	**0.0001**	**0.01**

R-values > 0.25 and p-value < 0.0005 indicate statistical significant discrimination among groups (highlighted in bold).

**Fig 2:**
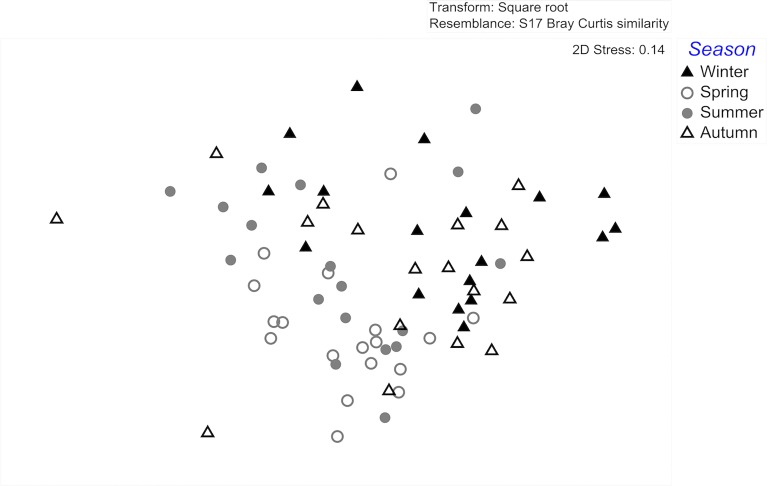
MDS (multi-dimensional scaling) plot of the variance in *Fucus serratus* surface extract composition originating from different seasons. Symbols represent single monthly *F*. *serratus* individuals within the four seasons (in all cases *n* = 18 per season).

The chemical composition of *Fucus serratus* surface extracts differed significantly among seasons (ANOSIM global test: global R = 0.293, *p* = 0.0001). The composition of winter extracts differed significantly from that of spring extracts (ANOSIM pairwise tests: winter/spring R statistic = 0.472, *p* = 0.0001) and summer extracts (ANOSIM pairwise tests: winter/summer R statistic = 0.338, *p* = 0.0001). Spring extracts differed significantly from autumn surface extracts (ANOSIM pairwise tests: spring/autumn R statistic = 0.425, *p* = 0.0001) (Table 2 and Fig 2).

**Table 2:** Pairwise test results (ANOSIM) for *Fucus serratus* chemical composition of surface extracts.

*Groups*	*R statistic*	*p-value*	*Significance level %*
**Winter, Spring**	**0.472**	**0.0001**	**0.01**
**Winter, Summer**	**0.338**	**0.0001**	**0.01**
Winter, Autumn	0.129	0.007	0.7
Spring, Summer	0.198	0.0006	0.06
**Spring, Autumn**	**0.425**	**0.0001**	**0.01**
Summer, Autumn	0.208	0.0007	0.07

R-values > 0.25 and p-value < 0.0005 indicate statistical significant discrimination among groups (highlighted in bold).

These statistical differences are clearly discernable in the MDS representation (Figs 1 and 2).

### Relationship between surface chemistry composition and environmental variables

The abiotic environmental variables (light intensity, seawater temperature and nutrient concentrations) recorded at the sampling site as well as the prokaryotic fouling pressure followed a seasonal cycle typical for Northern Germany. The surface seawater temperatures reached minimum values at the end of January and maximum values at the end of July. The light intensity increased from March onwards and reached peak intensities in August. The nutrient concentrations reached their minimum during the spring/summer months, followed by increasing concentrations in the autumn and winter months. Abiotic parameters are published in [34]. Prokaryotic *in situ* fouling pressure increased from April onwards and reached peak intensities in August [35].

The distance-based linear model (DistLM) analysis detected significant correlations between the surface chemistry composition of *Fucus* and the environmental variables (Table 3 and Table 4).

**Table 3:** Results of distance-based linear model (DistLM). Relationship between *Fucus vesiculosus* surface chemistry composition and the predictor variables. Model output contains only variables of the best fit.

*Variable*	*Adj*. *R*^*2*^	*SS(trac)*	*Pseudo-F*	*P*	*Prop*.	*Cumul*	*res*.*df*
**+ Light**	**0.4475**	1607.5	9.9115	**0.0005**	0.4977	0.4977	10
+ Nitrogen	0.4971	293.26	1.9866	0.1147	9.0811E-2	0.5885	9

Light and nitrogen data from [34] (doi:10.1594/PANGAEA.858055).

**Table 4:** Results of distance-based linear model (DistLM). Relationship between *Fucus serratus* surface chemistry composition and the predictor variables. Model output contains only variables of the best fit.

*Variable*	*Adj*. *R*^*2*^	*SS(trace)*	*Pseudo-F*	*P*	*Prop*.	*Cumul*.	*res*.*df*
**+ Light**	**0.2167**	770.99	4.0435	**0.0097**	0.2879	0.2879	10
**+ Temperature**	**0.3244**	426.66	2.5944	**0.0419**	0.1593	0.4472	9
+ Phosphate	0.3582	230.25	1.4738	0.2207	8.5987E-2	0.53325	8
+ Fouling	0.3874	205.96	1.3811	0.2378	7.6915E-2	0.61016	7

Temperature, light and phosphate data from [34] (doi:10.1594/PANGAEA.858055). Fouling data from [35] (doi:10.1594/PANGAEA.864067).

The sequential tests of the distance-based linear model revealed that the combination of light and nitrogen had the highest explanatory power for *Fucus vesiculosus* surface chemistry, together explaining 58.9% (49.7% adj. R^2^) of the variance (Table 3).

**Fig 3:**
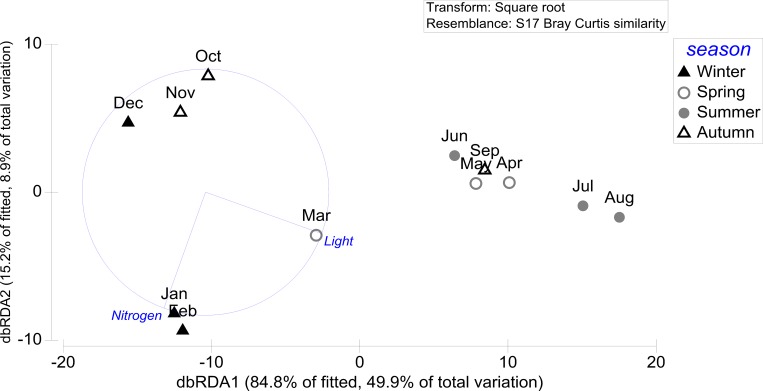
dbRDA plot (distance-based redundancy analysis) of the distance-based linear model (DistLM) based on the two predictor variables (light and nitrogen) fitted to the variance in *Fucus vesiculosus* surface chemistry composition. Light and nitrogen data from [34] (doi:10.1594/PANGAEA.858055).

The distance-based redundancy (dbRDA) plot illustrates the separation of the surface chemistry samples along the first and second axis correlating with the most important variable light on the first axis and with the variable nitrogen on the second axis. The variation on the first axis mainly discriminates spring and summer extract samples from autumn and winter samples (Fig 3). Light correlates with the first axis, which explains 49.8% of the variation in chemical composition. Nitrogen correlates with the second axis which explains 8.9% of the variation in chemical composition (Fig 3).

For *Fucus serratus*, the sequential tests of the distance-based linear model shows that the combination of all four environmental variables (light, temperature, phosphate and fouling) has the highest relevance, together explaining 61.01% (38.7% adj. R^2^) of the variance of *F*. *serratus* surface chemistry (Table 4).

**Fig 4:**
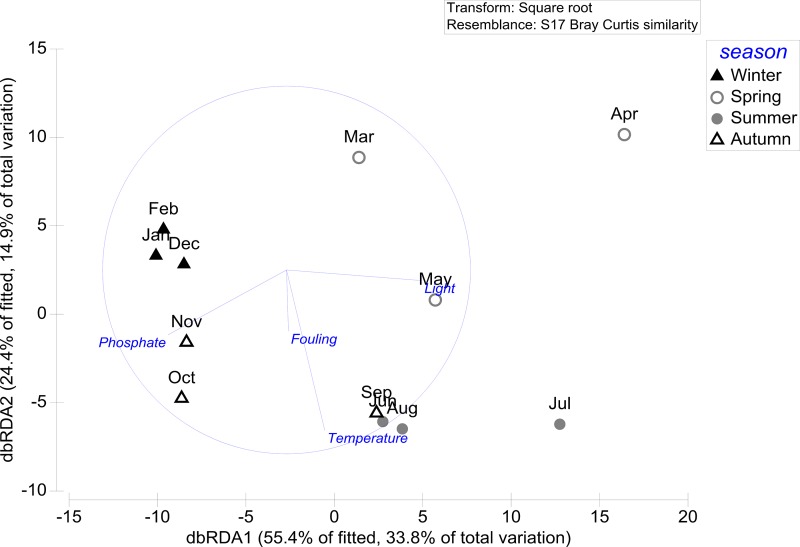
dbRDA plot (distance-based redundancy analysis) of the distance-based linear model (DistLM) based on the four predictor variables (temperature, light, phosphate and fouling) fitted to the variance in *Fucus serratus* surface chemistry composition. Temperature, light and phosphate data from [34] (doi:10.1594/PANGAEA.858055). Fouling data from [35] (doi:10.1594/PANGAEA.864067).

The dbRDA ordination plot shows that the two most important variables light and phosphate correlate with the first axis which explains 33.8% to the variation in chemical composition. Along the first axis, light and phosphate are negatively correlated to each other, resulting in a distinct grouping of mainly winter and autumn extract samples from summer and spring samples (Fig 4). Temperature and prokaryotic fouling correlate with the second axis which explains 14.9% to the variation in chemical composition (Fig 4).

### Contribution of surface metabolites to seasonal differences

A comparison of *F*. *vesiculosus* winter and summer surface extract composition revealed that two main signal groups (retention time 13–14 and 20–23 min, resp.) dominated by carbohydrates exhibited a pronounced up-regulation in summer extracts. Mono- and disaccharides were the prevalent up-regulated molecules in summer surface extracts. Furthermore, two different hydroxy acids were found to be up-regulated: citric acid and hydroxypropanoic acid. Citric acid was only present in summer surface extracts, whereas hydroxypropanoic acid was present during both seasons with a 7.7 and 5-fold up-regulation, respectively, in summer extracts compared to winter extracts (Table 5).

**Table 5:** Changing levels of metabolites in summer and winter surface extracts of *F*. *vesiculosus* from SIMPER analysis. Metabolites are ranked by regulation strength (log ratio).

Metabolite or class	Mass	Rt (min)	Winter (av. abund.)	Summer (av. abund.)	Ratio *(summer/winter)*	Log ratio *(summer/winter)*	Contrib. (*%*)
Citric acid**	273.1	14.29	< 0.0001*	0.24	2400	**3.38**	0.81
**Monosacch.**	117.1	13.60	0.19	< 0.0001*	0.0005	**-3.28**	0.63
**Disacch.**	205.1	21.90	< 0.0001*	0.14	1400	**3.15**	0.46
**unknown**	97.1	23.54	< 0.0001*	0.11	1100	**3.04**	0.36
**Disacch.**	204.1	21.89	0.01	0.23	23	**1.36**	0.74
**Disacch.**	117.0	20.43	0.01	0.22	22	**1.34**	0.69
**Monosach.**	319.2	14.95	0.01	0.16	16	**1.20**	0.49
**Disacch.**	217.1	21.88	0.01	0.15	15	**1.18**	0.48
**Monosacch.**	245.1	14.31	0.01	0.12	12	**1.08**	0.37
**Disacch.**	75.0	20.89	0.01	0.11	11	**1.04**	0.35
**Disacch.**	103.1	21.89	0.01	0.11	11	**1.04**	0.34
**Monosacch.**	205.1	14.95	0.02	0.17	8.50	**0.93**	0.51
**Disacch.**	204.1	21.13	0.02	0.17	8.50	**0.93**	0.48
Hydroxy-propanoic acid**	117.1	5.90	0.03	0.23	7.67	**0.88**	0.75
**Disacch.**	273.0	20.44	0.02	0.15	7.50	**0.88**	0.45
**Monosacch.**	205.1	13.03	0.05	0.36	7.20	**0.86**	1.04
**Disacch.**	363.2	20.51	0.07	0.44	6.29	**0.80**	1.21
**Disacch.**	361.2	20.45	0.19	1.16	6.11	**0.79**	3.19
**Disacch.**	217.1	20.46	0.14	0.85	6.07	**0.78**	2.37
**Disacch.**	361.2	21.13	0.06	0.35	5.83	**0.77**	0.98
**Monosacch.**	103.1	14.74	0.03	0.17	5.67	**0.75**	0.46
**Disacch.**	231.1	20.49	0.03	0.16	5.33	**0.73**	0.46
**Unknown**	131.1	7.57	0.04	0.2	5.00	**0.70**	0.54

Mass = gas chromatography–mass spectrometry mass output (can correspond to fragment ion after derivatization); Rt = retention time; av. abund. = average abundance derived from the relative peak area; Contrib. % = contribution in % to the dissimilarity between winter and summer group; < 0.0001* = original value was 0, transformed to calculate the ratio and log ratio; Monosacch. = Monosaccharide; Disacch. = Disaccharide

** co-injection with derivatised standards.

Comparing *F*. *serratus* winter and summer surface extract composition shows that two main signal groups (retention time 13–17 and 20–28 min) dominated by saturated fatty acids were up-regulated in summer surface extracts. Hexadecanoic acid (palmitic acid) and octadecanoic acid (stearic acid) were only present in summer surface extracts. Pentadecanoic and docosanoic acids were present in both seasons with a 4.5 and 5-fold up-regulation, respectively, in summer extracts compared to winter extracts (Table 6). Further, two carbohydrates were found to be up-regulated in summer extracts compared to winter extracts, whereby the detected disaccharide was 18-fold up-regulated comparing winter and summer extracts (Table 6).

**Table 6:** Changing levels of metabolites in winter and summer surface extracts of *F*. *serratus* from SIMPER analysis. Metabolites are ranked by regulation strength (log ratio).

Metabolite or class	Mass	Rt (min)	Winter (av. abund.)	Summer (av. abund.)	Ratio *(summer/winter)*	Log ratio *(summer/winter)*	Contrib. (%)
Hexadecanoic acid / FA**	129.0	16.15	< 0.0001*	0.09	900.0	**2.95**	0.48
Octadecanoic acid / FA**	341.2	17.66	< 0.0001*	0.06	600.0	**2.78**	0.35
**unknown**	204.1	28.41	< 0.0001*	0.05	5.0	**2.70**	0.28
**Disacch.**	204.1	21.86	0.02	0.36	18.0	**1.26**	2.06
**unkown**	149.0	19.69	0.01	0.13	13.0	**1.11**	0.74
Pentadecanoic acid / FA**	299.2	15.05	0.02	0.1	5.0	**0.7**	0.48
Docosanoic acid /FA**	129.0	20.36	0.02	0.09	4.5	**0.65**	0.50
**Sugar derivate/ Sacch.**	263.1	13.03	0.02	0.09	4.5	**0.65**	0.42

Mass = gas chromatography–mass spectrometry mass output (can correspond to fragment ion after derivatization); Rt = retention time; av. abund. = average abundance derived from the relative peak area; Contrib. % = contribution in % to the dissimilarity between winter and summer group; < 0.0001* = original value was 0, transformed to calculate the ratio and log ratio; FA = Fatty acid; Disacch. = Disaccharide; Sacch. = Saccharide

** co-injection with derivatised standards.

## Discussion

The perennial macroalgae *Fucus vesiculosus* and *Fucus serratus* are known to exhibit a seasonally variable chemical control of micro- and macrofoulers, with a tendency of stronger fouling reduction strength during seasons of high fouling pressure [13, 15, 34]. Therefore, it seems reasonable to assume that the chemical metabolite composition at the interface, which approaching foulers are first confronted with, is also not static but rather seasonally variable. To investigate this issue, the main focus of the present study lay on the variation in seasonal composition of surface metabolites, independently for *F*. *vesiculosus* and *F*. *serratus*.

Our study revealed that both *Fucus* species exhibited significant differences in surface chemistry composition between the seasons. Striking differences in surface metabolite composition were found between the two seasonal groups summer/spring (“summer”) and winter/autumn (“winter”). Specifically, a pronounced up-regulation of mono- and disaccharides and hydroxy acids in *F*. *vesiculosus* and up-regulated saccharides and fatty acids in *F*. *serratus* were found in summer surface extracts compared to winter extracts. Light was identified as the environmental variable with the highest explanatory power regarding the seasonal variance of the surface metabolite composition in both *Fucus* species.

### Contribution of surface metabolites to seasonal differences

*F*. *vesiculosus* summer and winter surface extract analysis revealed an up-regulation of mono- and disaccharides, citric acid as well as hydroxypropanoic acid in summer extracts compared to winter surface extracts. Our findings of up-regulated mono- and disaccharides match with previous results, which show that many macroalgae, including fucoids, exude large amounts of photosynthates (up to 30% of total fixed carbon) as dissolved organic carbon (DOC). This latter mainly consists of carbohydrates such as the monosaccharide glucose [4, 29, 40–42]. Sieburth [29] demonstrated that the exudation of organic matter in *F*. *vesiculosus* is directly coupled to photosynthesis and increases with increasing solar radiation. Additionally, it has been shown that the DOC release by many different species of macroalgae (from kelp to green algae) exhibits seasonal variation correlated to light availability and temperature and is synchronized with growth and photosynthetic rates [2, 4, 43]. These findings are supported by our results which show that light has the strongest and temperature the second strongest explanatory power of the seasonal shifts in *Fucus* surface metabolite composition. Since mono- and disaccharides, especially the monosaccharide glucose, function as ubiquitous energy sources from bacteria to humans, the observed up-regulation of mono- and disaccharides on *Fucus* surfaces should entail a profouling effect on the microbial fouler pool during summer months [13].

Beside saccharides, the hydroxy acids citric and hydroxypropanoic acid were found to be up-regulated in *F*. *vesiculosus* summer surface extracts compared to winter extracts. Citric acid or citrate, the conjugated base of citric acid, is the first intermediate product of the citric acid cycle in all aerobic organisms that involves the oxidative breakdown of organic molecules for energy generation and provision of intermediate products for biosynthesis. Therefore, it seems reasonable to assume that the pronounced up-regulation of citric acid could be connected to higher metabolic turn-over of *Fucus* during summer months. Hydroxypropanoic acid has been found in most brown and red algae as well as in low concentrations in green algae [44–46]. For both detected hydroxy acids antimicrobial activities have been reported, mainly from surveys with a medical or food technological background [47–50], and, further, an enhanced antimicrobial effect was found by mixing citric and maleic acids [48]. It is, thus, conceivable that these organic acids function as antibacterial agents on the thallus surface, reducing microbial densities. This assumption is supported by the fact that *Fucus vesiculosus* “summer” surface extracts originating from the same habitat exhibited strongest repelling effects against prokaryotic settlement when tested at near-natural concentration by means of *in situ* bioassays [35]. An antifouling effect obviously depends on the *in situ* surface concentrations of these acids and on the species-specific sensitivity of the various bacterial strains. The latter aspect of differential sensitivities may contribute to the “gardening” of biofilms and, ultimately, to the host-specificity of macroalgae-associated biofilms [51].

*Fucus serratus* summer surface extracts showed an up-regulation of two saccharides as well as of different fatty acids (FA). The dominant presence of FA among up-regulated metabolites in summer extracts is not exceptional, since marine macroalgae are rich in FA [52–54], with hexadecanoic acid or palmitic acid being the most common saturated FA in many macroalgae (21–42% of all fatty acids; [55]. Many FA have antimicrobial effects [56–58]. Palmitic acid, for instance, has antibacterial activity against different bacterial strains, including mycobacteria [59, 60]. The up-regulation of saccharides in *F*. *serratus* surface extracts is in accordance with the findings from *F*. *vesiculosus* and can be similarly interpreted (see previous paragraph).

*Fucus*, and macroalgae in general, do not exist in an axenic state in nature, but rather in a holobiont-like system tightly associated with a diverse community comprising mainly prokaryotes, fungi and diatoms [1, 51, 61]. Consequently, the analysed *Fucus* surface extracts harvested by the dipping extraction technique represent the combined surface metabolome of *Fucus* and its associated micro-epibionts. Seasonal variability in the holobiont composition would, accordingly, also be reflected in our metabolomic and ecologic investigation.

### Role of environmental variables for seasonal variation

Light had the strongest explanatory power for the seasonal fluctuations in surface metabolite composition, but temperature also contributed significantly to this variance. Nitrogen, phosphate and prokaryotic fouling pressure had less explanatory power (DistLM analysis, sequential test).

The strong relationship between light and surface metabolite composition is not surprising, considering that the compounds up-regulated in summer are metabolites closely related to photosynthesis (saccharides) or to storage metabolites (fatty acids). Former studies observed that phenolic phlorotannins in the brown alga *Cystoseira tamariscifolia* [62] and the antifouling sesquiterpene caulerpenyne from *Caulerpa taxifolia* [30], exhibit annual cycles regulated by solar radiation, showing higher compound concentrations in months with greatest irradiance. This type of light-dependent metabolite production in macroalgae and their partial exudation in *Fucus* (actively by transport or passively by loss of integrity of surface cells) through its outer thallus surface as described for the pigment fucoxanthin [8, 10] or dissolved organic carbon [29] could be the main (i.e. statistically dominant) cause for the observed seasonal variance in surface metabolite composition. However, it should be taken into consideration that the most prominently regulated metabolites, saccharides and fatty acids, may mask less dominant but, possibly, very fouling-active metabolites such as citric acid or proline (see above).

*Fucus serratus* surface metabolite composition was also significantly influenced by temperature. This relation could be indirect, since photosynthesis is also controlled by temperature [24]. Temperature influences the activities of several key enzymes of carbon metabolism such as the ribulose-1.5-bisphosphate carboxylase oxygenase (RuBisCO) [63, 64] as well as physical processes such as diffusion and carbon fixation. Typically, photosynthetic performance increases with increasing temperature up to a species-specific temperature maximum [24, 65–67]. The consequence, apparently, is a higher release of metabolites such as organic acids or carbohydrates into the diffusive boundary layer on the thallus surface (from where we extracted them).

Nitrogen and phosphate availability showed no significant influence on the chemical surface composition of either *Fucus* species. This non-significant relationship between nutrient availability and the surface metabolite composition is surprising, considering the fact that nutrients are known to modify the metabolism of plants [68]. In particular, dissolved nitrogen is known to favour photosynthesis, since nitrogen is essential for protein synthesis, and many key carbon assimilatory enzymes such as ribulose-1.5 bisphosphate carboxylase oxygenase (RuBisCO) as well as chlorophyll [69] and, hence, photosynthetic rates are dependent on nitrogen availability [70]. It has been demonstrated that elevated nutrient concentrations (NH_4_^+^, NO_3_^-^, PO_4_^3-^) enhance photosynthetic efficiency (again, when other factors are not stressful, [26]) and that accumulated tissue nitrogen could be the primary factor for the concentration of phenolic compounds in *F*. *vesiculosus* [71]. The lack of a significant relationship between nutrient availability and surface metabolite composition in the present study may be attributable to the fact that many macroalgae, including *F*. *vesiculosus*, have the ability to use internal nitrogen reserves for metabolic performances such as growth during seasons of nitrogen deficiency [72–74]. Therefore, it seems reasonable that the metabolism of both *Fucus* species was probably not nitrogen or nutrient-limited during our survey (August 2012—July 2013). Our findings regarding light and nutrients show similarities with the results from Pavia and Toth [28]. The authors reported that nitrogen availability has low explanatory power regarding the variation in tissue phlorotannin content of *F*. *vesiculosus*, whereas light exhibited greater importance in predicting the phlorotannin variability.

The seasonal variation on prokaryotic fouling pressure in the vicinity of *Fucus* did not relate directly and in real time to the surface metabolite variability in both *Fucus* species. This suggests that the variance in environmental microfouling pressure (assessed as number of cells settling per unit time on an artificial reference substratum) did not substantially drive or trigger the changes in surface chemistry on neighbouring *Fucus*. Previous studies [10, 32, 33], in contrast, showed that various macroalgae, including *F*. *vesiculosus*, exhibit a chemical antifouling control tuned to microbial fouling pressure. Given that the outer thallus surface represents the algal interface for all interactions with the environment and that bacterial epibionts are of primary importance to the wellbeing of their (algal) hosts [1] the absence of such correlation in our study is surprising. Indicative of their ecological function, macroalgal defence metabolites are typically concentrated in the outer meristoderm layers [75] or in specialised cells located at the thallus surface [76]. One possible explanation for the apparent and unexpected independence of fouling pressure and deployed defenses could be the following. Some surface-associated anti-microfouling compounds of *F*. *vesiculosus* are found in very small concentrations on the thallus, within the lower ng to μg-range (e.g. proline 0.09–0.59 ng cm^-2^; dimethylsulphopropionate (DMSP) 0.12–1.08 ng cm^-2^; fucoxanthin 0.7–9 μg cm^-2^) [9, 10]. Therefore, in our metabolomic approach these very minor chemical signals could have been masked by the strong compositional changes attributable to abundant photosynthesis-associated metabolites in the remaining mixture of surface-associated metabolites.

In conclusion, we demonstrate seasonal changes in surface chemistry on two *Fucus* species. A mixture of saccharides, hydroxy acids as well as fatty acids with potential pro- and antifouling properties comprise the up-regulated summer surface metabolites in *F*. *vesiculosus* and *F*. *serratus* originating from *Fucus* itself and, possibly, from its associated biofilm community. Although we could not link prokaryotic fouling pressure and surface chemistry in this study, our results show the importance of seasonal variation in surface chemistry as a factor for explaining macroalgae interactions with their environment that correlates with irradiance and temperature. Identifying the source and function of up-regulated metabolites will further enhance our understanding on the link between the *Fucus* surface metabolome and its interaction with potential foulers.
